# Supplementary material for: Antioxidant Responses of Phenolic Compounds and Immobilization of Copper in Imperata cylindrica, a Plant with Potential Use for Bioremediation of Cu Contaminated Environments
Source: Plants (Basel). 2020 Oct 20;9(10):1397. doi: 10.3390/plants9101397 (PMC7589974; doi:10.3390/plants9101397)
Supplement: Supplementary file 1 [file plants-09-01397-s001.pdf]

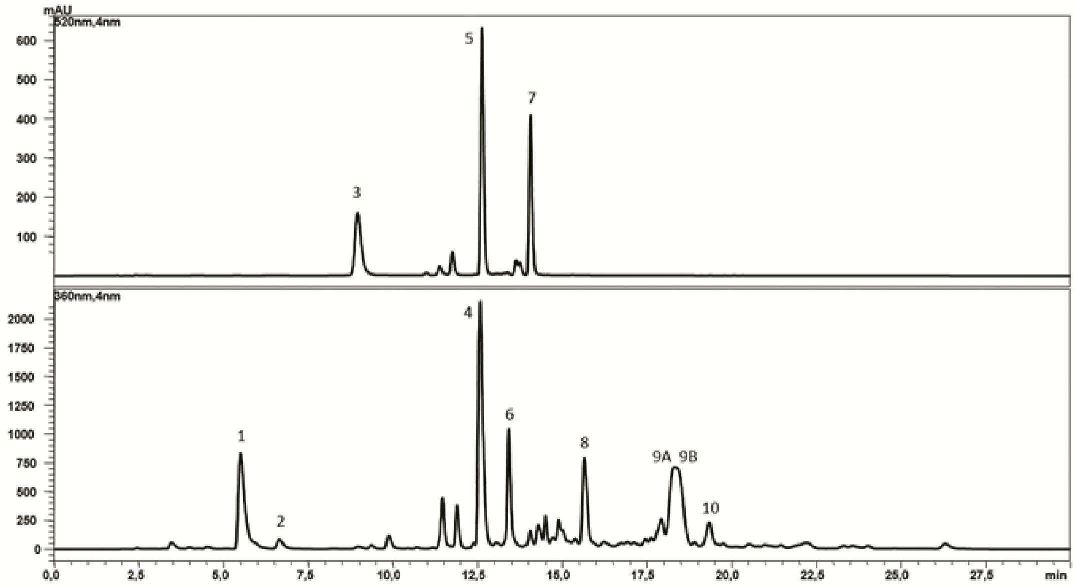

Supplementary Materials Figure S1. HPLC-DAD chromatogram of phenol compounds from *Imperata cylindrica* shoots after 21 days of exposure to Cu. **(A)** Wavelength 520 nm. **(B)** Wavelength 360 nm, where: 1) 5-caffeoylquinic acid, 2) caffeoylquinic acid isomer, 3) cyanidin-3-hexoside, 4) orientin, 5) cyanidin-3-malonylglucoside, 6) not identified, 7) cyanidin-derivative, 8) not identified, 9A) not identified, 9B) not identified, 10) not identified.
